# Data on complete genome sequence and annotation of two multidrug resistant atypical enteropathogenic *Escherichia coli* O177 serotype isolated from cattle faeces

**DOI:** 10.1016/j.dib.2022.108167

**Published:** 2022-04-14

**Authors:** Peter Kotsoana Montso, Victor Mlambo, Collins Njie Ateba

**Affiliations:** a Food Security and Safety Niche Area, Faculty of Natural and Agricultural Sciences, North-West University – Mafikeng Campus, Private Bag X2046, Mmabatho 2735, South Africa; b Bacteriophage Therapy and Phage Bio-Control Laboratory, Department of Microbiology, Faculty of Natural and Agricultural Sciences, North-West University– Mafikeng Campus, Private Bag X2046, Mmabatho 2735, South Africa.; c School of Agricultural Sciences, Faculty of Agriculture and Natural Sciences, University of Mpumalanga, Private Bag X11283, Mbombela 1200, South Africa.

**Keywords:** Escherichia coli O177, Whole genome sequence, Genome annotation, Genomic data, Virulence and Antimicrobial resistance genes

## Abstract

Atypical enteropathogenic *E. coli* belonging to the serotype O177 is a rare strain found in ruminants, especially cattle. When compared to shiga toxin producing *E. coli* (STEC) O157 and non-O157 STEC (O26, O45, O103, O104, O111, O121, and O145) serotypes, the antimicrobial resistance, virulence factors, and genomic structure of *E. coli* O177 are poorly understood. Therefore, in this article, we present the whole genome sequence data of two aEPEC *E. coli* O177 isolates (*E. coli* O177_CF-154-A and *E. coli* O177_CF-335-B) generated using Illumina MiSeq platform. The raw data were generated, cleaned, and assembled using Trimmomatic and SPAdes. Genome data analysis yielded 5,112,402 and 5,460,435 bp, comprising contigs 101 and 191 with GC contents of 50.7% and 50.5% for *E. coli* O177_CF-154-A and *E. coli* O177_CF-335-B, respectively. Prokaryotic Genome Annotation Pipeline (PGAP) and Rapid Annotation using Subsystem Technology (RAST) showed that the complete genome of *E. coli* O177_CF-154-A contained 5040 coding sequences (CDS), 5146 genes, 4896 proteins, 90 RNAs, and 78 tRNA while that of *E. coli* O177_CF-335-B contained 5463 CDS, 5570 genes, 5230 proteins, 92 RNAs, and 80 tRNA for. A total of 426 and 425 subsystem features with 5190 and 5662 CDS were obtained for *E. coli* O177_CF-154-A and *E. coli* O177_CF-335-B, respectively. Several genes encoding virulence and antimicrobial resistance were identified in both genomes. Complete genome sequence data of both isolates have been deposited in the National Center for Biotechnology Information (NCBI), GenBank: accession numbers, VMKH00000000 (*E. coli* O177_CF-154-A) and VMKG00000000 (*E. coli* O177_CF-335-B). This data can be used as a reference for determining the virulence and antimicrobial resistance in *E. coli* O177 isolates from different sample sources.

## Specifications Table

**Table:** 

Subject	Microbiology
Specific subject area	Molecular Microbiology and Bioinformatics
Type of data	TableFiguresExcel Sheets
How the data were acquired	Whole genome sequence was performed using Illumina MiSeq platform. The FASTQ files were obtained and imported into Kbase platform (https://kbase.us/). The files were subjected to FASTQC (v.0.11.5) to assess reads quality. Subsequently, raw data were processed using Trimmomatic (v0.36). The assemble algorithm was carried out using SPAdes (v3.13.0), and genome annotation was performed using Prokaryotic Genome Annotation Pipeline (PGAP), Rapid Annotation using Subsystem Technology (RAST) and Pathosystems Resource Integration Center (PATRIC).
Data format	Raw, filtered and analysed.
Description of data collection	Genomic DNA was extracted from two aEPEC O177 isolates (CF-154-A and CF-334-B) obtained from the Department of Microbiology, at NWU. The gDNA was sequenced using Illumina MiSeq platform. After sequencing FASTQ files were obtained. Raw reads were cleaned and assembled into contigs using FASTQC (v.0.11.5) SPAdes (v3.13.0), respectively. The genome annotation was carried out using PGAP, v.2.0 and RAST (v.2.0). The genome maps were drafted using PATRIC (v.3.6.2).
Data source location	• Institution: North-West University• City/Town/Region: North-West Province• Country*:* South Africa
Data accessibility	Repository name: National Center for Biotechnology Information (NCBI), GenBank, and figshare.Data identification numbers: VMKH00000000 (*E. coli* O177_CF-154-A) and VMKG00000000 (*E. coli* O177_CF-335-B);PRJNA555014 and PRJNA554852, SAMN12288806 and SAMN12285021*E. coli* O177_CF-154-A and for *E. coli* O177_ CF-335-B, respectively).Direct URL to data: https://www.ncbi.nlm.nih.gov/nuccore/VMKH00000000, https://www.ncbi.nlm.nih.gov/nuccore/VMKG00000000, https://figshare.com/s/e3a60e4a3d918527b572
Related research article	P. K. Montso, C. C. Bezuidenhout, C. Mienie, Y. M. Somorin, O. A. Odeyemi, V. Mlambo, C. N. Ateba*.* Genetic diversity and whole genome sequence analysis data of multidrug resistant atypical enteropathogenic *E. coli* O177 strains: An assessment of food safety and public health implications*. Int J Food Microbiol.* 2022, https://doi.org/10.1016/j.ijfoodmicro.2022.109555.

## Value of the Data

• These data provide genomic features of *E. coli* O177 serotype. Moreover, these data give an extensive information on the virulence and antimicrobial resistance profile of this serotype, which may contribute to understanding and improving of scientific knowledge of this pathogenic strain.
• The data may be used by researchers to develop new methods for detection of *E. coli* O177 serotype from different environmental samples. In addition, these data can be used in public health to establish policy framework and strategy intended to curb antimicrobial resistance, especially in humans.
• This genome can be used as a reference, especially for comparative genomic and epidemiological studies.

## 1 Data Description

**Fig 1:**
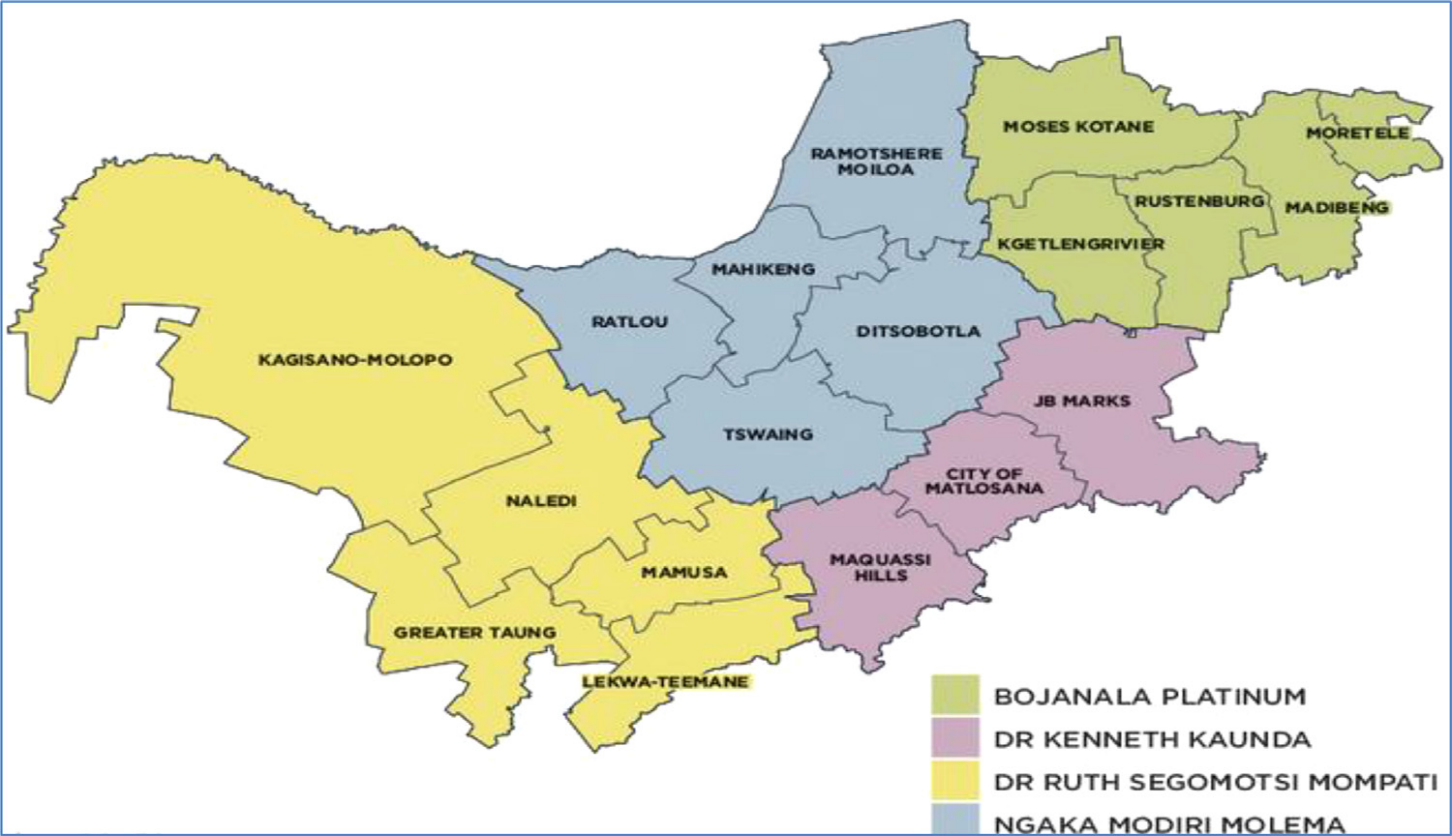
An illustration of the North West province map. https://municipalities.co.za/provinces/view/8/north-west.

**Fig. 2:**
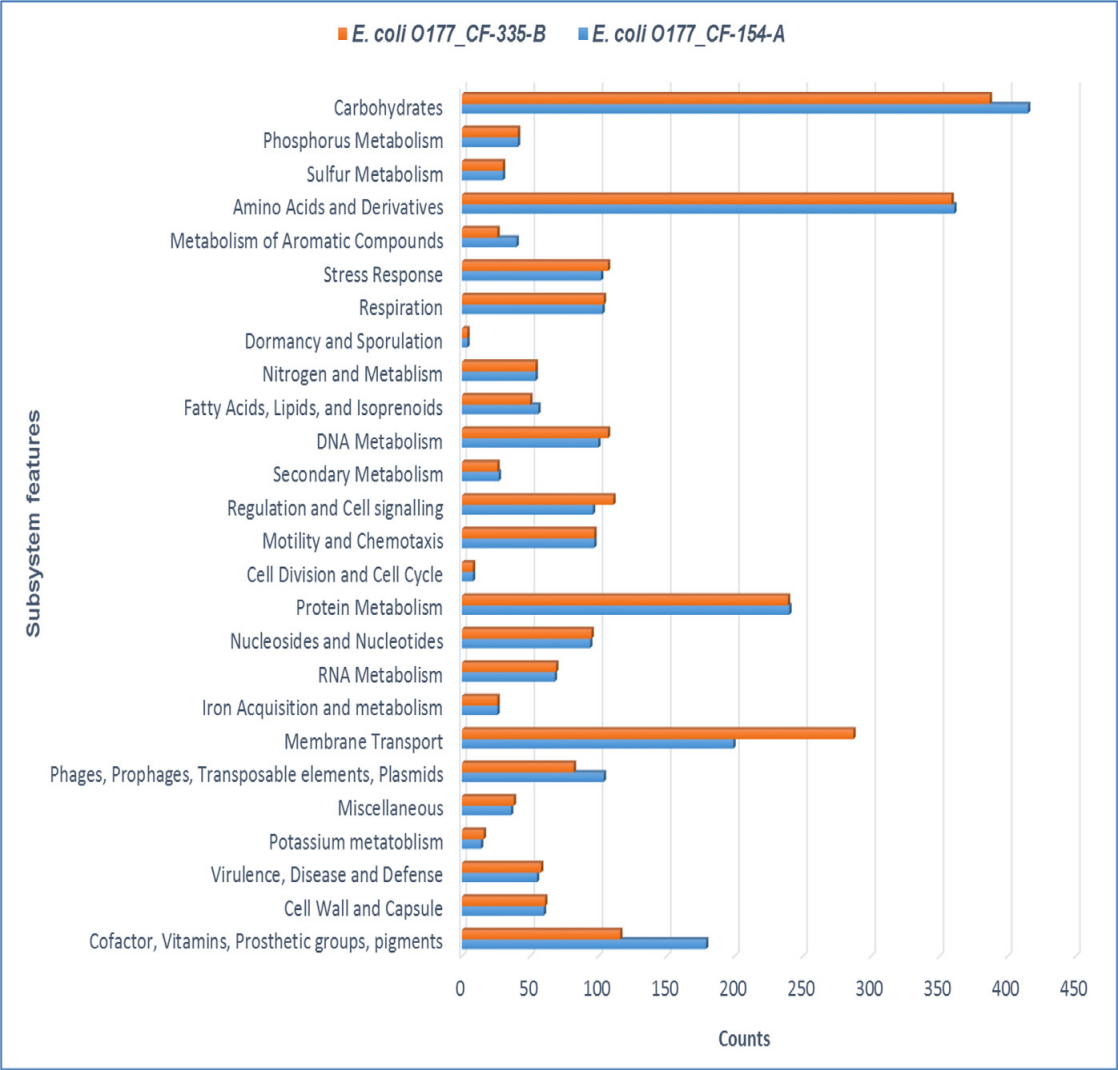
Frequency distribution of gene categories in genomes of two *E. coli* O177isolates obtained from cattle faeces.

**Fig. 3:**
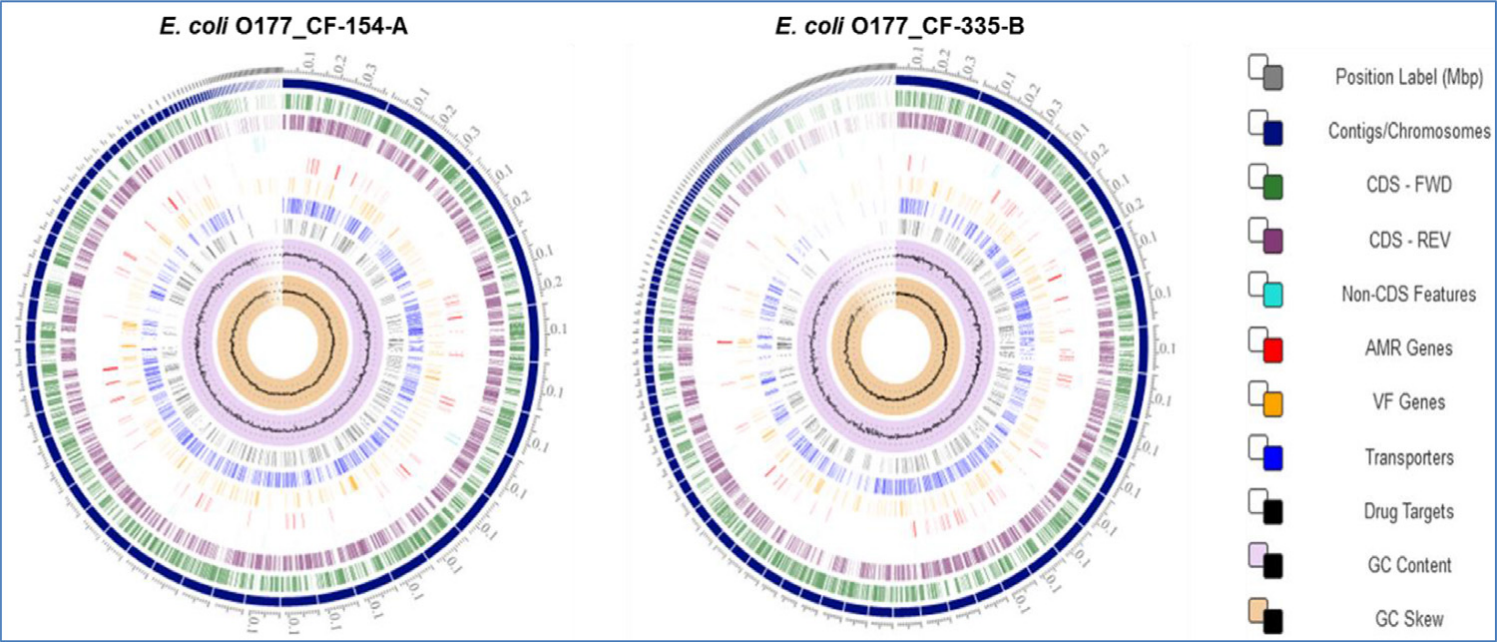
The circular genome map of *E. coli* O177 isolates (CF-154-A and CF-335-B) obtained from cattle faeces. Circle displays from inside to outside: GC Skew (light orange), GC content (light purple), Drug Tagets (black), Transporters (blue), Virulence factor genes (yellow), Antimicrobial resistance genes (red), Non CDS features (turquoise blue), CDS reverse strand (light purple) and CDS forward strand (green).

**Fig. 4:**
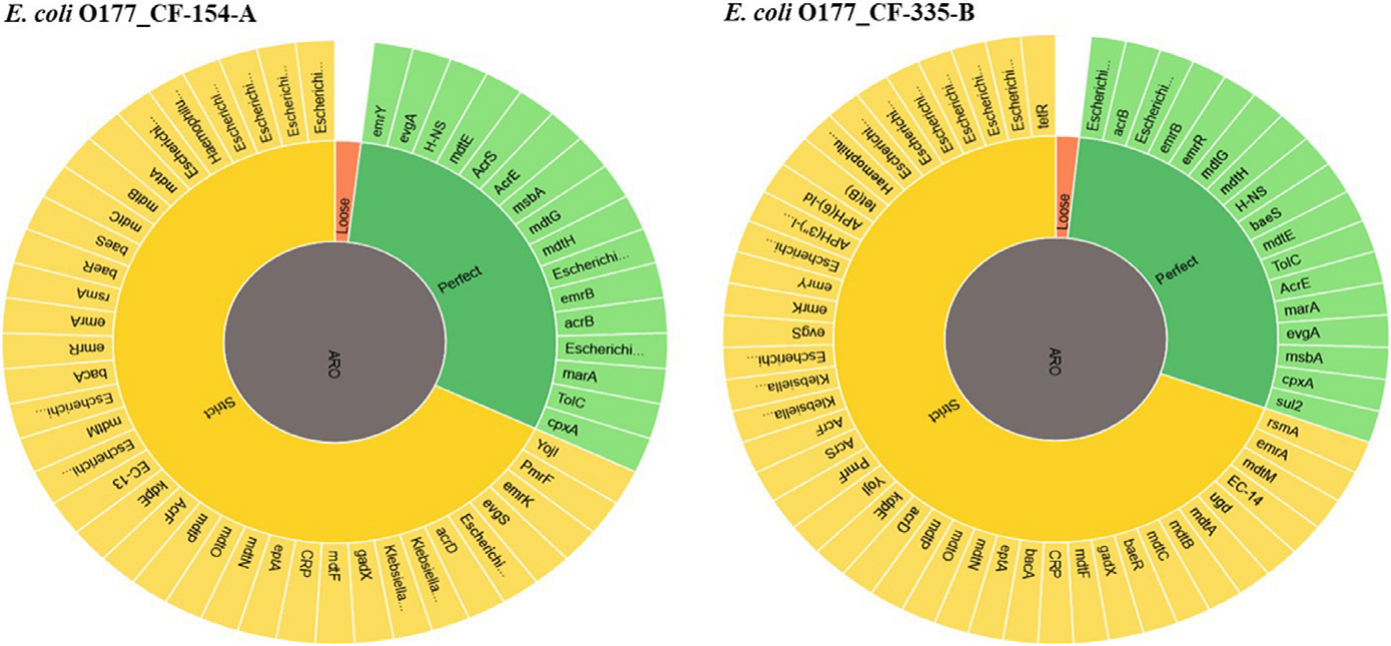
Distribution of antimicrobial resistance genes in genomes of two *E. coli* O177 isolates obtained from cattle faeces.

**Fig. 5:**
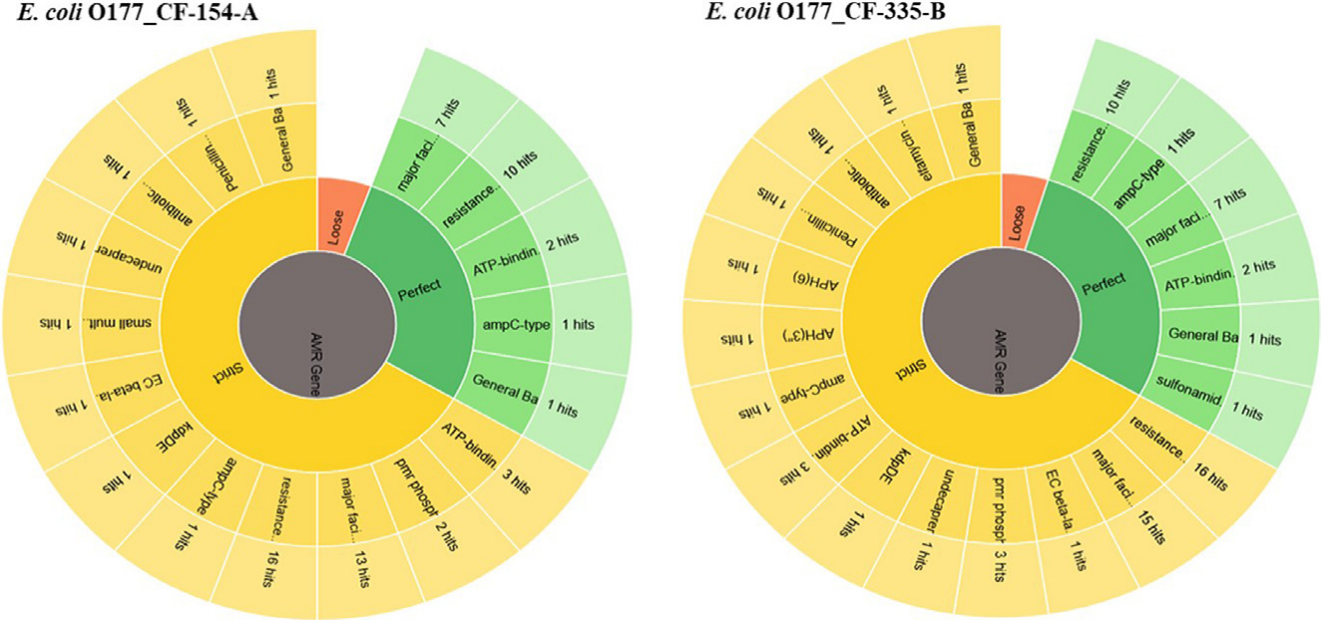
Distribution of antimicrobial resistance gene family in genomes of two *E. coli* O177 isolates obtained from cattle faeces.

**Fig. 6:**
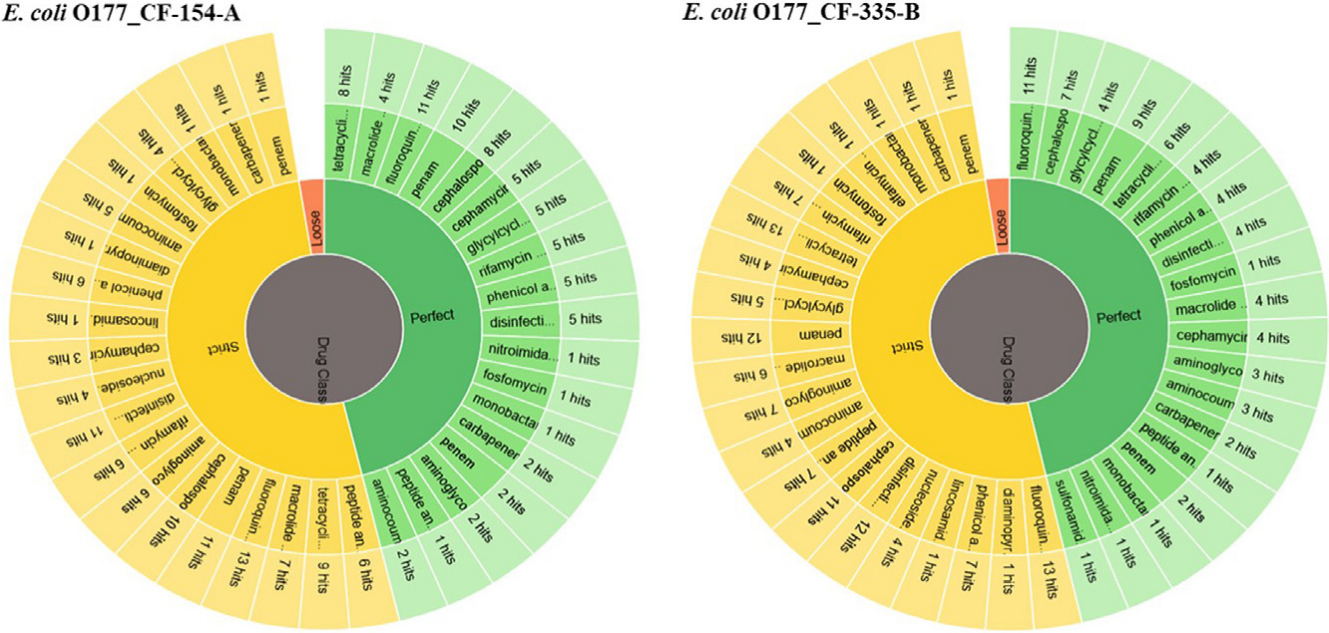
Antimicrobial drug classes in genomes of two *E. coli* O177 isolates from cattle faeces

**Fig. 7:**
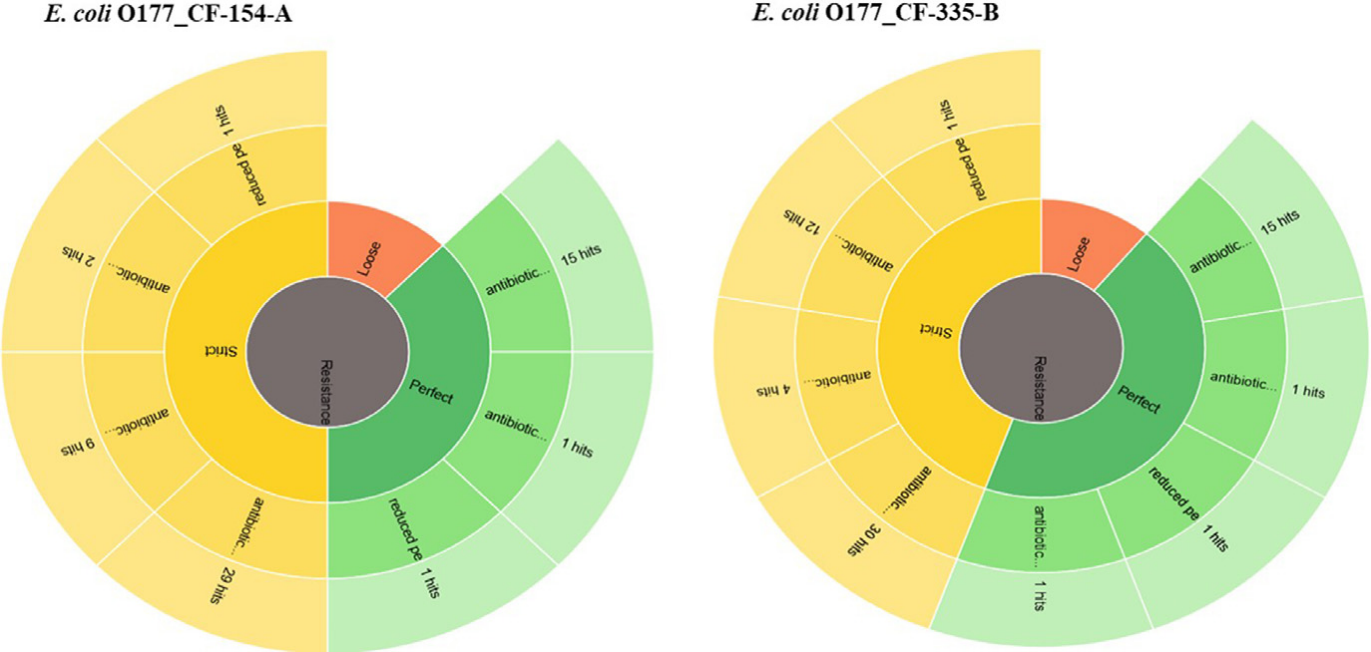
Resistance mechanisms in two *E. coli* O177 isolates from cattle faeces

Two atypical enteropathogenic *E. coli* O177 isolates (*E. coli* O177_CF-154-A and *E. coli* O177_CF-335-B) were obtained from cattle faeces in the North West province, South Africa (−27° 00′ 0.00″ S 26° 00′ 0.00″ E), Fig. 1. Genome sequencing was performed using Illumina MiSeq platform and a total of 576.5 Mb (CF-154’s genome) and 794.3 Mb (CF-335’s genome) raw data were obtained. The genome characteristics of the two isolates (*E. coli* O177_CF-154-A and *E. coli* O177_CF-335-B) are summarised in Table 1 and Fig. 2. The genome sizes were 5,112,402 and 5,460,435 bp, comprising contigs 101 and 191 with GC content of 50.7% and 50.5% for *E. coli* O177_CF-154-A and *E. coli* O177_CF-335-B, respectively. There were 5040 coding sequences (CDS), 5146 genes, 4896 proteins, 90 RNAs, and 78 tRNA for *E. coli* O177_CF-154-A genome, while *E. coli* O177_CF-335-B genome contained 5463 CDS, 5570 genes, 5230 proteins, 92 RNAs, and 80 tRNA. Furthermore, both genomes contained 2 CRISPR Arrays. Based on RAST annotation, there were 426 and 425 subsystem feature counts with 5190 and 5662 CDS in *E. coli* O177_CF-154-A and *E. coli* O177_CF-335-B, respectively. As depicted in Fig. 2, the carbohydrates; amino acids and derivatives; stress response; respiration; DNA metabolism; protein metabolism; membrane transport; and cofactor, vitamins, prosthetic groups, pigments were the most abundant subsystem feature found in both genomes. The circular complete genome draft shown in Fig 3 was constructed using CGView [1]. The Virulence and Resistance Gene Identifier revealed that both genomes contained several virulence and antimicrobial resistance genes, Figs 3-7 and Excel sheets 1 and 2 (S 1 and 2).

**Table 1:** Features of draft genomes of two *E. coli* O177 isolates obtained from cattle faeces.

	Sample ID	
Features	*E. coli* O177_CF-154-A	*E. coli* O177_CF-335-B
Genome size	5,112,402 bp	5,460,435 bp
Genome coverage depth	124.7x	162.128x
Total length	5111092 bp	5459908 bp
GC content (%)	50.7	50.5
Number of contigs	101	191
Contigs N50	127249	113919
Contigs L50	14	15
Number of Scaffold	101	-
Scaffold N50	130301	-
Scaffold L50	13	-
Coding genes	4896	5230
Total genes	5146	5570
Total CDSs	5040	5463
Total proteins	4896	5230
rRNA	8, 4, 6 (5S, 16S, 23S)	7, 4, 6 (5S, 16S, 23S)
tRNA	78	80
ncRNA	10	10
CRISPR Arrays	2	2

## 2 Experimental Design, Materials and Methods

### 2.1 Bacterial strain

Two atypical enteropathogenic *E. coli* O177 isolates were obtained from Antimicrobial Resistance and Phage Biocontrol Laboratory, Department of Microbiology. The isolates were selected based on the virulence and antimicrobial resistance profiles as described in the previous studies [2,3]. The stock cultures were removed from −80 °C and revived on MacConkey agar. The plates were incubated at 37 °C for 24 hours. After incubation, a single colony was transferred into 15 falcon tubes containing 10 mL nutrient broth. The tubes were incubated in a shaking incubator (150 rpm) at 37 °C for 24 hours.

### 2.2 Genomic DNA extraction and Sequencing

Genomic DNA was extracted from overnight cultures using the Zymo Research Genomic DNA^TM^-Tissue MiniPrep Kit (Biolab, South Africa) following the manufacturer's instructions. The DNA concentration was determined using the NanoDrop^TM^-Lite 1,000 spectrophotometer (Thermo Fisher Scientific, Walton, ma, USA). After fragmentation, DNA libraries were constructed using the Nextera XT DNA library prep kit (Illumina, USA) following the manufacturer's instruction. The fragmented DNA was amplified using 12 cycles PCR, which adds the index sequences [index 1 (i7) and index 2 (i5)]. The PCR products were purified using 0.6 × Agencourt AMPure XP beads (Beckman Coulter), and the quality was determined using 1.5% (w/v) agarose gel. Each library was diluted to 12 pmol. Samples were normalized to 4 nM using Nextra XT Library Normalization Beads (Illumina). Normalized libraries were pooled and 150 base paired-ends sequencing was performed with MiSeq Reagent V3 600-cycle kits on the Miseq instrument (Illumina).

### 2.3 Genome assembly, annotation and data analysis

Raw sequence data were generated and FASTQ files were obtained. The data were assessed for quality using FASTQC (v.0.11.5) and filtered for low quality reads and adapter regions using Trimmomatic (v.0.36) [4,5]. The *de novo* genome assembly was carried out using SPAdes (v.3.13) [5]. Complete genome annotation was performed using NCBI PGAP (v.5.0), Prokka pipeline (v.2.1.1), RAST server (v.2.0) and PATRIC online sever (v.3.6.2) [6], [7], [8], [9], [10]. Antimicrobial resistance genes were further mined using the Resistance Gene Identifier online tool of the comprehensive Antibiotic Resistance Database CARD^4^ (https://card.mcmaster.ca/analyze/rgi) with all parameters (‘Perfect and Strict hits’ and ‘High quality/Coverage’) set at default [11].

## Ethics Statements

This study did not involve the use of human subjects or animal experiments.

## CRediT authorship contribution statement

**Peter Kotsoana Montso:** Conceptualization, Methodology, Data curation, Writing – original draft, Visualization, Investigation, Software, Validation, Writing – review & editing. **Victor Mlambo:** Conceptualization, Methodology, Supervision, Software, Validation, Writing – review & editing. **Collins Njie Ateba:** Conceptualization, Methodology, Supervision, Software, Validation, Writing – review & editing.

## Declaration of Competing Interest

The authors declare that they have no known competing financial interests or personal relationships that could have appeared to influence the work reported in this paper.

## Data Availability

Data on Complete Genome Sequence and Annotation of Two Multidrug Resistant Atypical Enteropathogenic Escherichia coli O177 Serotype Isolated from Cattle Faeces (Original data) (Figshare). Draft Genome Sequence Data of Two Multidrug Resistant Atypical Enteropathogenic Escherichia coli O177 isolates obtained from Cattle faeces (Reference data) (Mendeley Data).
